# Hospitalizations for coronavirus disease 2019: an analysis of the occurrence waves

**DOI:** 10.1038/s41598-024-56289-7

**Published:** 2024-03-09

**Authors:** Juliana Rodrigues Tovar Garbin, Franciéle Marabotti Costa Leite, Cristiano Soares Silva Dell’Antonio, Larissa Soares Dell’Antonio, Ana Paula Brioschi dos Santos, Luís Carlos Lopes-Júnior

**Affiliations:** 1 Graduate Program in Public Health at the Universidade Federal do Espírito Santo (PPGSC/UFES), Vitória, ES Brazil; 2 Instituto Federal de Educação, Ciência e Tecnologia do Espírito Santo (IFES Campus Vitória), Vitória, ES Brazil; 3 Secretaria de Estado da Saúde do Espírito Santo-SESA/ES, VItória, ES Brazil

**Keywords:** COVID-19, SARS-CoV-2, Hospitalization, Health care, Medical research, Risk factors

## Abstract

The pandemic has been characterized by several waves defined by viral strains responsible for the predominance of infections. We aimed to analyze the mean length of hospital stay for patients with COVID-19 during the first three waves of the pandemic and its distribution according to sociodemographic and clinical variables. This retrospective study used the notifications of patients hospitalized for COVID-19 in a Brazilian state during the period of the three waves of the disease as the data source. There were 13,910 hospitalizations for confirmed COVID-19 cases. The first wave was the longest, with 4101 (29.5%) hospitalizations, while the third, although shorter, had a higher number of hospitalized patients (N = 6960). The average length of stay in the hospital in all waves was associated with age groups up to 60 years old., elementary, high school and higher education, residents of the periurban area Regarding the presence of comorbidities, there was a statistically significant difference in the mean number of days of hospitalization among patients with chronic cardiovascular disease and obesity (*P* < 0.001). In conclusion, the COVID-19 pandemic has been distinctly revealed among the waves.

## Introduction

Brazil was the first country in South America to confirm a case of coronavirus disease 2019 (COVID-19) on February 26, 2020. This occurred weeks after countries in the Northern Hemisphere, such as the United States, disclosed the same information^1,2^. However, the country had already declared in advance, on February 3, 2020, a Public Health Emergency of National Importance^3^, while the World Health Organization (WHO) recognized the disease as a Public Health Emergency of International Concern (PHEIC) at the end of January 2020^4^ and shortly thereafter as a pandemic. The pandemic has reached frightening limits, with greater concentration in the American and European region; by September 16, 2022, more than 600 million confirmed cases and 6 million deaths had been registered. Data show that many countries have presented four types of waves^5^.

Eighteen months after the emergence of the public health emergency, deficiencies caused by the overload of the Brazilian health system and its social and economic impacts were already felt in the states and capitals. A Brazilian study identified that in the first two waves, the highest incidence rates were found in the northern states of the country, where there is greater vulnerability: Amapá, Rio Grande do Norte, Rondônia, and Roraima. Amazonas and Rondônia have the worst mortality rates^6^. In Argentina, it was observed that similarly, during the first wave, neighborhoods with higher percentages of households with unmet basic needs had a higher risk of mortality from COVID-19^7^.

The pandemic has been characterized by several waves defined by viral strains responsible for the predominance of infections^8^. The rapid spread of the new coronavirus, especially in the first waves, resulting in morbidity and mortality indicators at worrying levels, has challenged government officials regarding the need to contain the spread of the disease, in addition to the urgent development of strategies that point to the planning of care services for the population, whether primary, secondary, or tertiary^9^.

Initially, approximately a quarter of people diagnosed with COVID-19 had developed serious conditions; of these, 80% needed to be admitted to the intensive care unit (ICU)^10^. A recent study analyzing the survival of patients hospitalized with COVID-19 in the state of Espírito Santo, Brazil, found that the group of people aged 80 years or older who were smokers, obese, had chronic cardiovascular disease, chronic kidney disease, chronic neurological disease, and neoplasms were associated with a shorter survival time^11^. Thus, it was possible to observe that some priority groups had more severe outcomes, such as death, owing to the favorable characteristics of these events.

Official estimates indicate that approximately 81% of people affected by COVID-19 could be assisted in primary health care, 14% would need hospital care, and 5% would occupy ICU beds. However, the emergence of new strains overturned this initial theory since they caused an increase in hospitalization rates^12^.

Hence, the present study aimed to analyze the mean length of hospital stay for patients with COVID-19 during the first three waves and their distribution according to sociodemographic and clinical variables.

## Results

**Figure 1:**
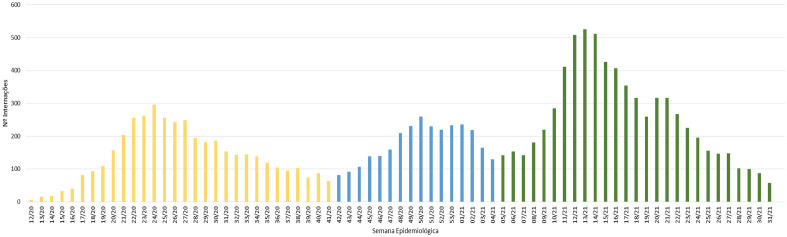
Number of hospitalizations due to COVID-19 according to the 1st, 2nd and 3rd wave.

During the established study period, it was noted that the first wave was the longest, with 30 epidemiological weeks and 4101 (29.5%) hospitalizations. The second had a shorter duration (16 EW) and a lower record of 2849 (20.5%) hospitalizations. However, the third showed a higher peak with 6960 (50%) hospitalizations, but it was shorter than the first wave, with 27 EW, as shown in Fig. 1.

The final number of hospitalizations obtained during the study period was 9017. As described in Table 1, most hospitalizations occurred in the third wave, with 4410 (48.9%), followed by the first wave with 2786 (30.9%), and the second wave with 1821 (20.2%) hospitalizations. However, the highest mean length of stay was observed in the first wave at 11.5 days (± SD 12.7). The second wave had a lower mean than that of the other two waves, at 10 days (± SD 10.1). The third wave presented an average greater than the second but smaller than the first, with 11.3 days (± 12.2). We observed a statistically significant difference when comparing the mean length of stay between the waves (*P* < 0.001).

**Table 1:** Distribution of hospitalizations due to COVID-19 according to the wave of occurrence.

Waves	Hospitalization count		Length of hospital stay for COVID 19 (Days)					P-value*
	n	%	Minimum	Maximum	Median	Mean	Standard deviation	
1	2786	30.90	2.0	133.0	8.0	**11.5b**	**12.7**	** < 0.001**
2	1821	20.20	2.0	114.0	7.0	10.0a	10.1	
3	4410	48.91	2.0	206.0	7.0	**11.3b**	**12.2**	

(*) Analysis of variance (ANOVA); ab—different letters indicate differences between means (Tukey's multiple comparison test); significant if p ≤ 0.050.

Significant values are in bold.

Regarding the sociodemographic characterization of hospitalized patients, as shown in Table 2, most of the sample was found in the first, second, and third wave, within the age group up to 59 years (47.8%, 44.6%%, and 58.5%), with elementary education (39.9%, 37.7%, and 43.03%), without pregnancy (98.33%, 97.02%, and 98.56%), race non-white color (60.28%, 53.52%, and 57.49%), male sex (54.7%, 53.8%, and 54.6%), with no disability (96.84%, 97.9%, and 98.4%), not being homeless (99.39%, 99.45%, and 99.55%), living in the urban area (94.07%, 90.95%, and 88.94%), not being a health professional (92.84%, 96.13%, and 97.93%), and not having a work-related infection (94.4%, 96.95%, and 98.55%).

**Table 2:** Characterization and association of sociodemographic variables between waves of occurrence, according to hospitalizations for COVID 19.

		Waves						P-values*
		1		2		3		
		n	%	n	%	n	%	
Age group	Up to 59 y.o	1333	47.85	812	44.59	2579	58.48	** < 0.001**
	60 to 79 y.o	1034	37.11	712	39.10	1434	32.52	
	> 80 y.o	419	15.04	297	16.31	397	9.00	
Education**	Illiterate	107	6.83	62	5.61	107	4.72	** < 0.001**
	Elementary	625	39.89	417	37.70	976	43.03	
	High school	513	32.74	393	35.53	831	36.64	
	Higher education	322	20.55	234	21.16	354	15.61	
Sex	Female	1263	45.33	841	46.18	2004	45.44	0.832
	Male	1523	54.67	980	53.82	2406	54.56	
Pregnant**	No	1233	98.33	813	97.02	1981	98.56	**0.018**
	Yes	21	1.67	25	2.98	29	1.44	
Race/color**	Non-White	1337	60.28	784	53.52	1996	57.49	** < 0.001**
	White	881	39.72	681	46.48	1476	42.51	
Person with disability**	No	2571	96.84	1783	97.91	4338	98.37	** < 0.001**
	Yes	84	3.16	38	2.09	72	1.63	
Homeless**	No	2608	99.39	1811	99.45	4390	99.55	0.665
	Yes	16	0.61	10	0.55	20	0.45	
Zone**	Urban	2521	94.07	1588	90.95	3732	88.94	** < 0.001**
	Rural	158	5.90	155	8.88	456	10.87	
	Periurban	1	0.04	3	0.17	8	0.19	
Health professionals**	No	2465	92.84	1641	96.13	3929	97.93	** < 0.001**
	Yes	190	7.16	66	3.87	83	2.07	
Work-related infection**	No	1651	94.40	1524	96.95	3610	98.55	** < 0.001**
	Yes	98	5.60	48	3.05	53	1.45	

(*) Pearson's chi-square test; significant if p ≤ 0.050.

(**) The remainder of the sample was within the “ignored” and “not applicable” categories.

Significant values are in bold.

Regarding comorbidities, according to the descriptive analysis, as shown in Table 3, a higher proportion was found in the first wave. Patients with chronic lung disease were present in 8.8% (245) in the first wave, 4.7% (86) in the second wave, and 3.7% (162) in the third wave. Similarly, followed by those with chronic cardiovascular disease (50.34%, 43.2%, and 35.07%), chronic kidney disease (3.9%, 2.2%, and 1.6%), chronic liver disease (0.8%, 0.4%, and 0.4%), diabetes mellitus (26%, 22%, and 16.7%), human immunodeficiency virus (HIV) infection (0.6%, 0.5%, and 0.2%), tuberculosis (0.4% in the first wave and 0.02 in the second), and neoplasms (2.9%, 2.5%, and 1.3), who were smokers (5.1%, 3.6%, and 2.1%), who underwent bariatric surgery (0.2%, 0.2%, and 0.2%), and were obese (12%, 9.3%, and 8.5%). Different from the others, patients with chronic neurological disease presented 5.4% (98) in the second wave, 5.1% (141) in the first wave, and 3.1% (138) in the third, and those with immunodeficiency represented 1% in the second, 0.9% in the first, and 0.5% in the third wave.

**Table 3:** Characterization and association of comorbidities between waves of occurrence, according to hospitalizations for COVID 19.

		Waves						P-values *
		1		2		3		
		n	%	n	%	n	%	
Chronic lung disease	No	2533	91.18	1732	95.27	4243	96.32	** < 0.001**
	Yes	245	8.82	86	4.73	162	3.68	
Chronic cardiovascular disease	No	1379	49.66	1031	56.74	2860	64.93	** < 0.001**
	Yes	1398	50.34	786	43.26	1545	35.07	
Chronic kidney disease	No	2669	96.08	1779	97.80	4333	98.39	** < 0.001**
	Yes	109	3.92	40	2.20	71	1.61	
Chronic liver disease	No	2756	99.21	1812	99.56	4387	99.59	0.078
	Yes	22	0.79	8	0.44	18	0.41	
Diabetes mellitus	No	2052	73.89	1418	78.00	3670	83.31	** < 0.001**
	Yes	725	26.11	400	22.00	735	16.69	
Immunodeficiency	No	2753	99.14	1802	99.01	4380	99.48	0.080
	Yes	24	0.86	18	0.99	23	0.52	
Human immunodeficiency virus infection	No	2760	99.39	1810	99.45	4392	99.75	**0.045**
	Yes	17	0.61	10	0.55	11	0.25	
Smoking	No	2635	94.89	1754	96.37	4311	97.89	** < 0.001**
	Yes	142	5.11	66	3.63	93	2.11	
Bariatric surgery	No	2765	99.78	1815	99.78	4398	99.84	0.813
	Yes	6	0.22	4	0.22	7	0.16	
Obesity	No	2437	87.98	1663	91.47	3996	90.74	** < 0.001**
	Yes	333	12.02	155	8.53	408	9.26	
Tuberculosis	No	2766	99.64	1820	100.00	4403	99.98	** < 0.001**
	Yes	10	0.36	0	0.00	1	0.02	
Neoplasms	No	2696	97.12	1774	97.47	4348	98.71	** < 0.001**
	Yes	80	2.88	46	2.53	57	1.29	
Chronic neurological disease	No	2636	94.92	1722	94.62	4265	96.87	** < 0.001**
	Yes	141	5.08	98	5.38	138	3.13	

(*) Pearson's chi-square test; significant if p ≤ 0.050.

Significant values are in bold.

The comparison of the average number of days of hospitalization for each category of sociodemographic variables showed significance for the following variables: age group, education, being pregnant, race/color, being homeless and the area of residence, where the age group up to 59 years old had similar averages between the first wave 1 (mean = 9.8 days; ± SD 10.7 days) and the third wave (mean = 10.1 days; ± SD 11.1 days). For the range of 60 to 79 years, the means were similar between the first wave (mean = 13.3 days; ± SD 14.0 days) and the third wave (mean = 13.3 days; ± SD 13.9 days). And for those over 80 years old, the means were similar between the first wave (mean = 12.7 days; ± SD 14.4 days) and the third wave (mean = 11.8 days; ± SD 12.0 days). When we look at the education variable, we notice that there was similarity in the average number of days of hospitalization for high school between the first wave (mean = 10.5 days; ± SD 10.1 days) and the third wave (mean = 11.4 days; ± SD 11.3 days). And for those with higher education, in the third wave had a higher average (mean = 14.0 days; ± SD 17.6 days). The same was observed for pregnant women in the third wave (mean = 8.1 days; ± SD 9.3 days). For white race/color, there was similarity in the mean number of days of hospitalization between the first wave (mean = 11.5 days; ± SD 12.4 days) and the third wave (mean = 11.2 days; ± SD 11.7 days). And for non-whites, the highest average was for the first wave of the pandemic (mean = 12.3 days; ± SD 13.7 days).

Regarding the homeless people, the highest average was for the second wave (mean = 17.3 days; ± SD 16.7 days). And for those living in urban areas, there was similarity in the average number of days spent in hospital between the first wave (mean = 11.5 days; ± SD 12.7 days) and the third wave (mean = 11.6 days; ± SD 12.3 days) (Table 4).

**Table 4:** Analysis of the average length of stay for COVID 19, according to wave of occurrence and sociodemographic variables.

		Waves						P-values*
		1		2		3		
		Length of hospital stay for COVID 19 (Days)						
		Mean	Standard deviation	Mean	Standard deviation	Mean	Standard deviation	
Age group	Up to 59 y.o	**9.8b**	**10.7**	7.9a	7.6	**10.1b**	**11.1**	** < 0.001**
	60 to 79 y.o	**13.3b**	**14.0**	11.4a	11.6	**13.3b**	**13.9**	** < 0.001**
	≥ 80 y.o	**12.7b**	**14.4**	12.0a	11.2	**11.8b**	**12.0**	** < 0.001**
Education	Illiterate	12.1a	12.1	10.9a	12.3	9.5a	8.9	0.223
	Elementary	12.8a	14.8	11.5a	10.2	11.7a	12.1	0.174
	High school	**10.5b**	**10.1**	8.6a	8.1	**11.4b**	**11.6**	** < 0.001**
	Higher education	10.5a	12.8	9.9a	9.0	**14.0b**	**17.6**	** < 0.001**
Sex	Female	**11.3b**	**12.9**	9.6a	9.3	**11.1b**	**12.0**	**0.003**
	Male	**11.8b**	**12.5**	10.3a	10.7	**11.5b**	**12.4**	**0.007**
Pregnant	Yes	4.1ab	2.5	3.5a	1.8	**8.1b**	**9.8**	**0.020**
Race/Color	Non-White	**11.5b**	**12.4**	9.7a	9.2	**11.2b**	**11.7**	**0.001**
	White	**12.3b**	**13.7**	10.3a	10.8	11.3ab	13.1	**0.007**
Person with disability	Yes	11.0a	10.0	14.7a	12.2	13.8a	15.6	0.239
Homeless	Yes	7.9a	4.4	**17.3b**	**16.7**	9.7ab	4.6	**0.028**
Zone	Urban	**11.5b**	**12.7**	9.9a	9.7	**11.6b**	**12.3**	** < 0.001**
	Rural	10.9a	11.9	10.5a	13.4	9.6a	11.8	0.440
	Periurban	44.0a	0.0	22.3a	18.0	24.0a	25.5	0.723
Health professionals	Yes	9.7a	11.4	10.7a	12.9	14.3a	27.0	0.121
Work-related infection	Yes	11.0a	14.4	10.6a	13.2	18.5a	34.0	0.080

(*) Analysis of variance (ANOVA); ab—different letters indicate differences between means (Tukey's multiple comparison test); significant if p ≤ 0.050.

Significant values are in bold.

Chronic cardiovascular disease, diabetes mellitus and chronic neurological disease showed differences in the average number of days of hospitalization between the waves, where the average number of days for chronic cardiovascular disease was higher in the first wave (mean = 13.3 days; ± SD 13.8 days). For diabetes mellitus it was in the first wave (mean = 13.9 days; ± SD 14.5 days) and for chronic neurological disease it was also in the first wave (mean = 16.7 days; ± SD 19.7 days) (Table 5). When analyzing the relationship between days of hospitalization due to COVID-19 in all waves with sociodemographic and clinical variables, the variables that were significant were age group, education, area of residence, chronic cardiovascular disease and obesity, where, in the age group of 60 years and over there was a tendency for an increase in the median number of hospitalizations compared to those in the age group of up to 59 years. Those with elementary, secondary and higher education also showed a median increase in the number of days spent in hospital compared to those who were illiterate. Those who live in rural and peri-urban areas have a longer median number of days in hospital compared to those who live in urban areas. Those who had chronic cardiovascular disease and obesity also showed an increasing trend in the median number of days spent in hospital compared to those who did not have these comorbidities (Table 6).

**Table 5:** Analysis of the average length of stay for COVID 19, according to wave of occurrence and comorbidities.

		Waves						P-values*
		1		2		3		
		Length of hospital stay for COVID 19 (days)						
		Mean	Standard deviation	Mean	Standard deviation	Mean	Standard deviation	
Chronic lung disease	Yes	13.4a	14.1	13.0a	11.7	14.2a	18.3	0.812
Chronic cardiovascular disease	Yes	**13.3b**	**13.8**	11.4a	11.2	12.5ab	12.3	**0.003**
Chronic kidney disease	Yes	13.7a	12.7	14.5a	11.7	13.4a	16.8	0.919
Chronic liver disease	Yes	14.9a	14.4	12.4a	7.4	12.7a	7.1	0.771
Diabetes mellitus	Yes	**13.9b**	**14.5**	11.3a	11.7	13.0ab	12.9	**0.008**
Immunodeficiency	Yes	12.9a	9.1	12.3a	10.7	18.1a	12.8	0.164
Human immunodeficiency virus infection	Yes	20.4a	22.5	12.3a	8.7	13.2a	19.9	0.480
Smoking	Yes	16.0a	18.1	12.3a	10.0	16.5a	20.7	0.286
Bariatric surgery	Yes	6.8a	5.2	5.8a	3.6	7.7a	6.3	0.846
Obesity	Yes	14.8a	16.5	11.6a	12.5	14.6a	16.5	0.088
Tuberculosis	Yes	11.3a	12.4	0.0a	0.0	11.0a	0.0	0.982**
Neoplasms	Yes	15.3a	14.3	13.4a	10.9	12.7a	13.1	0.492
Chronic neurological disease	Yes	16.7b	19.7	12.0a	11.3	12.8ab	10.7	**0.027**

(*) Analysis of variance (ANOVA); ab—different letters indicate differences between means (Tukey's multiple comparison test); significant if p ≤ 0.050.

(**) Student's t test for independent samples; significant if p ≤ 0.050.

Significant values are in bold.

**Table 6:** Association of the average length of stay for COVID 19 overall (all waves) with sociodemographic and clinical variables.

Dependent variable—length of hospital stay for COVID 19 (days)		Coefficient	Robust standard error	95% CI for Coefficient		P-values*	Trend
				Inferior limit	Upper limit		
Age group	Up to 59 y.o	0	–	–	–	–	–
	60 to 79 y.o	2	0.46	1.090	2.91	** < 0.001**	Increase
	≥ 80 y.o	2	0.76	0.510	3.49	**0.003**	Increase
Education	Illiterate	0	–	–	–	–	–
	Elementary	− 0.27	0.06	− 0.39	− 0.16	** < 0.001**	Decrease
	High school	− 0.58	0.06	− 0.70	− 0.46	** < 0.001**	Decrease
	Higher education	− 0.65	0.06	− 0.78	− 0.53	** < 0.001**	Decrease
Zone	Urban	0	–	–	–	–	–
	Rural	− 0.26	0.04	− 0.34	− 0.18	** < 0.001**	Decrease
	Periurban	0.60	0.11	0.38	0.81	** < 0.001**	Increase
Chronic cardiovascular disease	No	0	–	–	–	–	–
	Yes	0.44	0.03	0.38	0.5	** < 0.001**	Increase
Obesity	No	0	–	–	–	–	–
	Yes	− 0.17	0.04	− 0.24	− 0.1	** < 0.001**	Decrease

(*) Multiple quantile regression with the forward method; (0) reference category; significant if p ≤ 0.050.

Variables inserted in the model: Age group, education, sex, pregnant, race/color, person with disability, homeless, zone, health professionals, work-related infection, chronic lung disease, chronic cardiovascular disease, chronic kidney disease, diabetes mellitus, immunodeficiency, human immunodeficiency virus infection, smoking, bariatric surgery, obesity, tuberculosis, neoplasms, chronic neurological disease.

Significant values are in bold.

## Discussion

This study aimed to analyze the mean length of hospitalization for patients with COVID-19 during the first three waves and their distribution according to sociodemographic and clinical variables. Notably, the third wave of the COVID-19 pandemic had higher hospitalization rates in a short period of time, which makes it difficult to track and isolate infected cases^13^.

A study on the clinical manifestations of COVID-19 states that the clinical spectrum ranges from mild to moderate to severe, in which severe cases progress to acute respiratory distress syndrome (ARDS) and the need for admission to the ICU^14^. ARDS is characterized by the impairment of respiratory function secondary to a diffuse interstitial-alveolar inflammatory process of the lung parenchyma, causing significant edema and fibrosis^15^.

To analyze such variations, it must be considered that the material, human, and structural resources were different from one another. The virus has mutated; thus, the variants circulating at each moment of the pandemic were not the same, causing variations in clinical manifestations and case severity^16–18^.

The state of Espírito Santo followed a trend in the behavior of case records that had already occurred in Brazil. The first pandemic wave of this study recorded 2786 hospitalizations (30.9%), and in the second wave, 1821 hospitalizations (20.2%). However, in the third wave, the highest hospitalization rates were recorded, with 4410 hospitalizations (48.9%).

In the country, the first phase was marked by a continuous decrease in physical distancing measures, followed by a gradual increase in cases, hospitalizations, and deaths. The second phase of transmission began in the summer, the period of the year-end festivities and holidays, in addition to the easing of restriction measures, mainly in December 2020, with predominance of the Gama variant, with a peak in April 2021. The third wave began in December 2021, which coincided with the festive period, vacations, relaxation of measures, and introduction of the Omicron variant. In this period, the influenza A virus epidemic also appeared, increasing the number of severe acute respiratory syndrome (SARS) cases^19^. It is important to emphasize that despite the course of the pandemic and the increase in the number of hospital beds in the third wave, the critical levels of occupancy and strangulation of the care network were in several states of Brazil^17^.

Regarding the mean length of stay, there was a similarity between the first and third waves. The period of stay in the hospital was similar to another study conducted in the country, although without analysis among the waves^14^. Multiple factors can interfere with the length of stay of a patient. However, the clinical severity of the case and dependence on care are the main factors that are influenced by individual patient issues, such as age, sex, and clinical history of comorbidities^20^. There was a predominance of hospitalizations among people aged up to 79 years in all three waves. In addition, in the third wave of the pandemic, hospitalizations among people under 59 years of age reached 58.5%, a crucial moment of the pandemic where the resumption of economic activities was observed more intensely and, with that, the reduction in social distancing measures. The study pointed out a statistically significant difference in the average length of hospital stay among patients in the age groups up to 59 years and from 60 to 79 years.

Although the mortality from COVID-19 is higher among older adults, the Pan American Health Organization, on April 26, 2021, warned of the change in the age profile of hospitalized cases and those admitted to the ICU; there was a higher rate of hospitalization in the younger population globally, which corroborates our findings^21^. A study conducted in Salvador, Bahia, also showed an increase in the proportion of young adults without comorbidities and severe COVID-19 in the second wave^22^.

A study carried out in the state of Espirito Santo, Brazil, when estimating the years of potential life lost (YPLL), pointed out that deaths related to COVID-19 in younger people resulted in a greater number of YLLL in relation to the more advanced age groups. This fact, added to other indicators, reveal a decrease in life expectancy and losses in relation to the economically active population^23^.

Although there was a predominance of the sample among the lowest education levels, going in the same direction as other studies^11,24,25^. People from high school and higher education, specifically in the third wave, had this time increased, mainly among the higher education, whose average jumped from 10.5 days in the first wave to 14 days in the third, reflecting slower progress and worsening of cases. This finding may suggest a worsening scenario with an increasing number of infections, especially among the less vulnerable population. It is known that to improve its adaptation to population, climatic, and environmental factors, the dissemination and aggressiveness of new viral variants of SARS-CoV-2 is a consequence of its various mutations, translating its enormous capacity for survival and change^26^.

Pregnancy is characterized as a period with several physiological changes, mainly in the immune and cardiovascular systems, making it more susceptible to respiratory and systemic complications in viral infections. In the state of Espírito Santo, Brazil, COVID-19 in pregnancy had a higher risk of maternal death (relative risk [RR] 18.73–95% confidence interval [95%CI] 11.07–31.69), fetal death/stillbirth (RR 1.96–95%CI 1.18–3.25), preterm birth [RR 1.18–95%CI 1.01–1.39], cesarean delivery (RR 1.07–95%CI 1.02–1.11), and cesarean delivery occurring before the onset of labor (RR 1.33–95%CI 1.23–1.44)^27^.

Regarding the race/color variable, there was a higher record of hospitalizations in non-white people in all pandemic waves. However, both Caucasian and non-Caucasian patients had longer average hospital stays in both the first and third waves. In this context, we extend the relationship to the impact of the race/color variable, which is complex, as it is not limited to biological/genetic factors but represents a set of meanings and sociocultural exposures that portray inequity in health^28,29^.

Regarding sex, it is noted in the present study that males had the highest number of hospitalizations in the three waves. However, both sexes showed differences in the length of hospital stay during the waves. If women have an odds ratio of 1.43 (95% confidence interval [CI] = 1.04–1.96) for fatigue or muscle weakness compared with that of men, consequently leading to hospitalization^28^. On the other hand, literature data also indicate that being females reduced by 33% (Prevalence Ratio (PR) = 0.67, 95% CI = 0.62–0.71) the probability of being admitted to the ward and 38% (PR = 0.62, 95% CI = 0.56–0.69) in the ICU, and the probability of dying is 37% (PR = 0.63, 95% CI = 0.57–0.70) compared with that of males^24^. Some experts have not detected significant differences regarding the clinical course and mortality in the ICUs between the variant SARS-CoV-2 Alpha and WT between sexes^30^.

However, an ecological study with data on hospitalizations due to COVID-19 in women in the Unified Health System (SUS) observed that women with high levels of comorbidities associated with the main diagnosis of COVID-19 had a longer hospital stay^31^. Therefore, it is important to study the influence of biological factors such as sex on the clinical management of the disease^32^.

Patients with serious and complicated conditions due to COVID-19 usually require hospitalization in the ICU and may develop several limitations and sequelae, including post-intensive care syndrome^33,34^.

Although the pandemic has affected the country's population, its impact may differ due to existing inequalities. In the present study, being a resident of an urban area showed a statistical difference in the length of stay during the three waves of COVID-19. However, after the regression, it became clear that people living in rural and peri-urban areas had a longer median number of days in hospital compared to those living in urban areas. It is important to emphasize that characteristics related to urban geography and city planning may be associated with the speed of spread of the disease, especially in cities that presented difficulties in physical and social distancing measures^35^ that were applied in different proportions in different periods of the pandemic. In addition, it is important to consider that those with more precarious living and working conditions, , have greater difficulties in accessing essential goods and services, which may result in differences observed in variables, such as area of residence^36^. In Brazil, the illness profile was uneven, being portrayed in association between the confirmed diagnosis of COVID-19 and per capita income, suggesting a socioeconomic difference in access to the diagnosis of COVID-19^37^. The presence of comorbidities associated with the severity of COVID-19 has already been described in other studies^14,38^. In addition, comorbidity is known to increase the risk of death by 9.44%^39^. Among the comorbidities associated with the length of stay in this study, cardiovascular disease and obesity contributed to a higher average hospital stay in the three waves of COVID-19, especially in the first and third waves, corroborating the study by Lana^40^, in which chronic renal failure, diabetes mellitus, other chronic lung diseases, and cardiovascular disease were at increased risk of hospitalization and death.

In the state of ES, the presence of cardiovascular diseases represented more than half of the confirmed comorbidities (54.37%) in patients with COVID-19, followed by diabetes (19.95%) and obesity (9.34%), recording at the end of the second wave an incidence rate with an increasing trend (p < 0.05)^41^. Similarly, a study conducted in New York reported that hospitalized patients with COVID-19, who were severely obese, older adults, and male sex were independently associated with higher in-hospital mortality and worse hospital outcomes in general^42^.

When analyzing the presence of comorbidities in people hospitalized during the three waves of COVID-19, there was no decrease between the first and third waves. The large decrease in access to primary healthcare found in Brazil was caused by COVID-19, which may have been an important factor for the non-reduction of cases associated with comorbidities in the SUS. The decline in the number of consultations, tests, and surgeries was seen in the third wave, with an increase in deaths from illnesses other than COVID-19, such as myocardial infarction, cancer, and stroke^12^.

Some limitations of the present study should be considered, such as that the data came from a secondary bank. The incompleteness of the variables was important, especially those related to the patient's hospitalization, which was responsible for the exclusion of a large part of the sample. It is known that inconsistencies in a large flow of information can be common, especially during a pandemic period, where data need to be computed in real-time for a quick management response. In addition, there is a high turnover of professionals; therefore, the time available for training may have been impaired. Nevertheless, most findings are compatible with the global literature and can contribute to epidemiological analyses of the behavior of COVID-19 throughout the pandemic waves, extrapolating the State of Espírito Santo, Brazil.

This study represents the first analysis of hospitalizations due to COVID-19 in the State of Espírito Santo, Brazil, permeating the three waves of the pandemic. It was possible to identify the behavior of patients' length of hospital stay according to their sociodemographic and clinical characteristics. The understanding that the first and third waves of the COVID-19 pandemic had worse records, especially in those patients in the age groups up to 60 , with elementary, high school and higher education, living in a periurban area, with chronic cardiovascular disease, and obesity. These findings pointed out that the pandemic revealed itself distinctly between the waves. A thorough understanding of the context experienced allows the health system to perform a reflective analysis of the phases in which cases increase. Therefore, it is suggested that efforts should be invested in both professional training and active surveillance of cases, in care networks (particularly in primary health care), and in the management of post-COVID-19 syndromes.

## Materials and methods

### Study design and population

This retrospective study used the notifications of individuals hospitalized for COVID-19 in the state of Espírito Santo, Brazil, covering the period from March 16, 2020 to August 07, 2021, as a data source. The state of Espírito Santo is located in the southeastern region of Brazil, with a population of 4,108,508 inhabitants distributed across 78 municipalities. The data were obtained from the Espírito Santo State Department of Public Health (SESA-ES) through the e-SUS Health Surveillance Information System (e-SUS-VS).

### Study population

**Figure 2:**
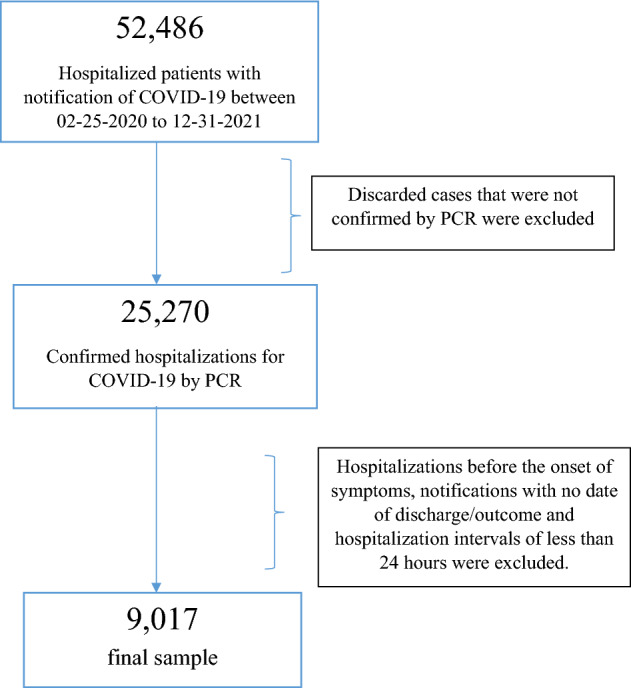
Flowchart for the patients selection process.

The study population consisted of all notifications for COVID-19 between the first2 years of the pandemic, from February 25, 2020 to December 31, 2021, resulting in a total of 52,986. After verifying laboratory confirmation, negative cases were excluded, leaving 25,270 cases confirmed by reverse transcription polymerase chain reaction (RT-PCR). Subsequently, a case curve was constructed in which the wave definition criterion was applied, which consisted of the beginning of an upward trend with significant evolution, reaching its plateau and tending to fall. Its end was marked by the lowest record of hospitalizations until the beginning of the next upward movement. To calculate the average number of days of hospitalization, it was also necessary to exclude those who had been hospitalized before the onset of symptoms, with no discharge/end date in the notification, and those who had a hospitalization interval of less than 24 h, totaling a number of 9017 hospitalizations analyzed. Figure 2 depicts the selection process of the research participants.

### Eligibility and outcomes

The wave of hospitalizations due to COVID-19 was defined as the beginning of an upward trend with significant evolution, reaching a plateau and tending to fall, with its end being marked by the lowest record of hospitalizations until the beginning of the next ascension movement. The notifications of patients hospitalized for COVID-19 during the three waves of the disease in Espírito Santo were used as a data source. The inclusion criteria include the confirmation of COVID-19 by RT-PCR and hospitalization in Espírito Santo. Thus, based on these criteria, it was established that the first wave occurred between epidemiological weeks (EW) 12 and 41 of 2020 (March 16, 2020 to October 10, 2020), the second wave between weeks 42 of 2020 and 4 of 2021 (October 11, 2020 to January 30, 2021), and the third wave between weeks 5 and 31 of 2021 (January 31, 2021 to August 8, 2021).

Subsequently, for data analysis and calculation of the hospitalization time interval, hospitalizations before the onset of symptoms, absence of discharge/outcome date, and hospitalization interval of less than 24 h were selected as exclusion criteria.

### Statistical analysis

The programs used in the analyzes were IBM SPSS Statistics version 26 and STATA version 15.1 (StataCorp, College Station, TX, USA).

The description was presented by frequency, percentage, minimum and maximum values, measures of central tendency and variability.

Analysis of variance (ANOVA) with Tukey's multiple comparison test and Student's T test compared the average number of days of hospitalization between waves. It was not necessary to use the normality test, because, according to Bussab and Morettin^43^, even if the data does not follow a normal probability distribution, when there is a very large independent and identically distributed sample, it approximates a normal distribution.

Pearson's chi-square test associated the waves with sociodemographic and clinical variables.

Multiple quantile regression with robust standard error and forward variable selection method related the time in days of hospitalization due to COVID-19 with sociodemographic and clinical variables. The advantages of this regression pointed out by Koenker and Bassett^44^ are: it is required when the distribution is not Gaussian (normal); is robust to outliers; when the residuals are not normal and/or non-homoscedastic, they produce more efficient estimators than those of ordinary least squares (OLS) regression and are more informative, not only being restricted to an average, as regression can be obtained using the median.

The alpha level of significance used in all analyzes was 5%.

### Ethical considerations

Ethical approval was obtained from the Research Ethics Committee of the Health Sciences Center of the Federal University of Espírito Santo (approval number 5.180.941) on December 20, 2021. We followed the ethical standards set by the Helsinki Declaration of 1964, revised in 2013 and the research guidelines of the Federal University of Espírito Santo. The Research Ethics Committee has considered the collection of routine data as an evaluation of service, as well as being a retrospective study, and therefore, waived the need for written informed consent. Additionally, we followed the Strengthening the Reporting of Observational Studies in Epidemiology (STROBE) guidelines^45^ (Supplementary Information).

### Supplementary Information

Supplementary Information.

## Data Availability

The datasets used and/or analysed during the current study available from the corresponding author on reasonable request.

## Abbreviations

CI: Confidence interval;
COVID-19: Coronavirus disease 2019
WHO: World Health Organization
PHEIC: Public Health Emergency of International Concern
ICU: Intensive care unit
SARS: Severe acute respiratory syndrome
PR: Prevalence ratio
SESA-ES: Espírito Santo State Department of Public Health
e-SUS-VS: Surveillance Information System
RT-PCR: Reverse transcription polymerase chain reaction
