# Supplementary material for: Hospitalizations for coronavirus disease 2019: an analysis of the occurrence waves
Source: Sci Rep. 2024 Mar 9;14:5777. doi: 10.1038/s41598-024-56289-7 (PMC10924092; doi:10.1038/s41598-024-56289-7)
Supplement: Supplementary file 1 — Supplementary Information. [file 41598_2024_56289_MOESM1_ESM.docx]

**STATISTICAL ANALYSIS**

The programs used in the analyses were IBM SPSS Statistics version 26 and STATA version 15.1 (StataCorp, College Station, TX, USA). The description was presented by frequency, percentage, minimum and maximum values, measures of central tendency, and variability.

Analysis of variance (ANOVA) with Tukey's multiple comparisons test and the T-test compared the mean number of days of hospitalization between waves. The use of normality test was not necessary, as stated by Bussab and Morettin (2017), even if the data do not follow a normal probability distribution, when there is a very large independent and identically distributed sample, it approaches a normal distribution. The Pearson chi-square test associated the waves with sociodemographic and clinical variables.

Multiple quantile regression with robust standard error and forward variable selection method related the length of hospitalization for COVID-19 in days with sociodemographic and clinical variables. The advantages of this regression pointed out by Koenker and Bassett (1978) are: it is required when the distribution is not Gaussian (normal); it is robust to outliers; when the residuals are not normal and/or not homoscedastic, it produces more efficient estimators than ordinary least squares (OLS) regression and is more informative, not only restricted to a mean, as the regression can be obtained by the median.

The alpha level of significance used in all analyses was 5%.

For the relationship between COVID-19 hospitalization days and sociodemographic and clinical variables in Wave 2, there was a significant association with age group, residential zone, chronic lung disease, chronic cardiovascular disease, and neoplasms. In the age group of 60 years or older, there was a tendency for an increase in the median of hospitalizations in Wave 2 compared to those in the age group up to 59 years. Those residing in the peri-urban zone had a higher median hospitalization days compared to those living in the urban zone. Similarly, individuals with chronic lung disease, chronic cardiovascular disease, and neoplasms also showed a trend of an increase in the median of hospitalization days in Wave 2 compared to those without these comorbidities (**Supplementary Table 1**).

**Supplementary Table 1.** Association of average hospitalization time for COVID 19 in the second wave with sociodemographic and clinical variables.

| Dependent variable - Length of hospital stay for COVID 19 (Days) | | Coefficient | Robust standard error | 95% CI for Coefficient | | P-values * | Trend |
| --- | --- | --- | --- | --- | --- | --- | --- |
|  |  |  |  | Inferior limit | Upper limit |  |  |
| Age group | Up to 59 y.o. | 0 | - | - | - | - | - |
|  | 60 to 79 y.o. | 2 | 0.34 | 1.340 | 2.66 | **< 0.001** | Increase |
|  | ≥ 80 y.o. | 2 | 0.45 | 1.120 | 2.88 | **< 0.001** | Increase |
| Zone | Urban | 0 | - | - | - | **-** | - |
|  | Rural | 1 | 0.52 | -0.030 | 2.03 | 0.056 | No significant |
|  | Periurban | 14 | 3.6 | 6.940 | 21.06 | **< 0.001** | Increase |
| Chronic lung disease | No | 0 | - | - | - | - | - |
|  | Yes | 3 | 0.72 | 1.590 | 4.41 | **< 0.001** | Increase |
| Chronic cardiovascular disease | No | 0 | - | - | - | - | - |
|  | Yes | 1 | 0.32 | 0.380 | 1.62 | **0.002** | Increase |
| Neoplasms | No | 0 | - | - | - | - | - |
|  | Yes | 2 | 0.93 | 0.170 | 3.83 | **0.032** | Increase |

(*) Multiple quantile regression with the forward method; (0) reference category; significant if p ≤ 0.050

Variables inserted in the model: Age group, education, sex, pregnant, race/color, person with disability, homeless, zone, health professionals, work-related infection, chronic lung disease, chronic cardiovascular disease, chronic kidney disease, diabetes mellitus, immunodeficiency, human immunodeficiency virus infection, smoking, bariatric surgery, obesity, tuberculosis, neoplasms, chronic neurological disease.

For the relationship between COVID-19 hospitalization days and sociodemographic and clinical variables in Wave 3, there was a significant association with age group, education, residential zone, chronic cardiovascular disease, immunodeficiency, and obesity. In the age group of 60 years or older, there was a tendency for an increase in the median of hospitalizations in Wave 3 compared to those in the age group up to 59 years. Individuals with a high school and higher education also influenced a median increase in the number of hospitalization days compared to illiterate individuals. Those residing in the peri-urban zone had a higher median hospitalization days compared to those living in the urban zone. Similarly, individuals with chronic cardiovascular disease, immunodeficiency, and obesity also showed a trend of an increase in the median of hospitalization days in Wave 3 compared to those without these comorbidities (**Supplementary Table 2**)

**Supplementary Table 2.** Association of average hospitalization time for COVID 19 in the third wave with sociodemographic and clinical variables.

| Dependent variable - Length of hospital stay for COVID 19 (Days) | | Coefficient | Robust standard error | 95% CI for Coefficient | | P-values * | Trend |
| --- | --- | --- | --- | --- | --- | --- | --- |
|  |  |  |  | Inferior limit | Upper limit |  |  |
| Age group | Up to 59 y.o. | 0 | - | - | - | - | - |
|  | 60 to 79 y.o. | 2 | 0.46 | 1.090 | 2.91 | **< 0.001** | Increase |
|  | ≥ 80 y.o. | 2 | 0.76 | 0.510 | 3.49 | **0.003** | Increase |
| Education | Illiterate | 0 | - | - | - | - | - |
|  | Elementary | 1 | 0.93 | -0.830 | 2.83 | 0.283 | No significant |
|  | High school | 2 | 0.97 | 0.110 | 3.89 | **0.038** | Increase |
|  | Higher education | 3 | 1.04 | 0.970 | 5.03 | **0.004** | Increase |
| Zone | Urban | 0 | - | - | - | - | - |
|  | Rural | -1 | 0.61 | -2.200 | 0.2 | 0.103 | No significant |
|  | Periurban | 19 | 6.34 | 6.570 | 31.43 | **0.003** | Increase |
| Chronic cardiovascular disease | No | 0 | - | - | - | **-** | - |
|  | Yes | 1 | 0.44 | 0.140 | 1.86 | **0.022** | Increase |
| Immunodeficiency | No | 0 | - | - | - | **-** | - |
|  | Yes | 11 | 2.7 | 5.700 | 16.3 | **< 0.001** | Increase |
| Obesity | No | 0 | - | - | - | **-** | - |
|  | Yes | 3 | 0.58 | 1.870 | 4.13 | **< 0.001** | Increase |

(*) Multiple quantile regression with the forward method; (0) reference category; significant if p ≤ 0.050

Variables inserted in the model: Age group, education, sex, pregnant, race/color, person with disability, homeless, zone, health professionals, work-related infection, chronic lung disease, chronic cardiovascular disease, chronic kidney disease, diabetes mellitus, immunodeficiency, human immunodeficiency virus infection, smoking, bariatric surgery, obesity, tuberculosis, neoplasms, chronic neurological disease.

Overall (across all waves), the relationship between COVID-19 hospitalization days and sociodemographic and clinical variables was significant with age group, education, residential zone, chronic cardiovascular disease, and obesity. In the age group of 60 years or older, there was a tendency for an increase in the median of hospitalizations compared to those in the age group up to 59 years. Individuals with elementary, high school, and higher education also influenced a median increase in the number of hospitalization days compared to illiterate individuals. Those residing in rural and peri-urban zones had a higher median hospitalization days compared to those living in the urban zone. Similarly, individuals with chronic cardiovascular disease and obesity also showed a trend of an increase in the median of hospitalization days compared to those without these comorbidities (**Supplementary Table 3**).

**Supplementary Table 3.** Association of the average length of stay for COVID 19 overall (all waves) with sociodemographic and clinical variables.

| Dependent variable - Length of hospital stay for COVID 19 (Days) | | Coefficient | Robust standard error | 95% CI for Coefficient | | P-values * | Trend |
| --- | --- | --- | --- | --- | --- | --- | --- |
|  |  |  |  | Inferior limit | Upper limit |  |  |
| Age group | Up to 59 y.o. | 0 | - | - | - | - | - |
|  | 60 to 79 y.o. | 2 | 0.46 | 1.090 | 2.91 | **< 0.001** | Increase |
|  | ≥ 80 y.o. | 2 | 0.76 | 0.510 | 3.49 | **0.003** | Increase |
| Education | Illiterate | 0 | - | - | - | - | - |
|  | Elementary | -0.27 | 0.06 | -0.39 | -0.16 | **< 0.001** | Decrease |
|  | High school | -0.58 | 0.06 | -0.70 | -0.46 | **< 0.001** | Decrease |
|  | Higher education | -0.65 | 0.06 | -0.78 | -0.53 | **< 0.001** | Decrease |
| Zone | Urban | 0 | - | - | - | - | - |
|  | Rural | -0.26 | 0.04 | -0.34 | -0.18 | **< 0.001** | Decrease |
|  | Periurban | 0.60 | 0.11 | 0.38 | 0.81 | **< 0.001** | Increase |
| Chronic cardiovascular disease | No | 0 | - | - | - | - | - |
|  | Yes | 0.44 | 0.03 | 0.38 | 0.5 | **< 0.001** | Increase |
| Obesity | No | 0 | - | - | - | - | - |
|  | Yes | -0.17 | 0.04 | -0.24 | -0.1 | **< 0.001** | Decrease |

(*) Multiple quantile regression with the forward method; (0) reference category; significant if p ≤ 0.050

Variables inserted in the model: Age group, education, sex, pregnant, race/color, person with disability, homeless, zone, health professionals, work-related infection, chronic lung disease, chronic cardiovascular disease, chronic kidney disease, diabetes mellitus, immunodeficiency, human immunodeficiency virus infection, smoking, bariatric surgery, obesity, tuberculosis, neoplasms, chronic neurological disease.

**REFERENCES**

BUSSAB, W.O. e Morettin, P.A., Estatística Básica, Saraiva, São Paulo, 9 ed, 2017.

KOENKER, R. and Bassett, G. (1978). *Regression Quantiles*, Econométrica, Vol. 46, Nº1, pp. 33-50.
